# Use of N-Acetylcysteine in Preterm Neonates with Enteral Feeding Intolerance and Intestinal Obstruction: A Case Series and Review of the Literature

**DOI:** 10.3390/children11070873

**Published:** 2024-07-18

**Authors:** Domenico Umberto De Rose, Francesca Landolfo, Flaminia Pugnaloni, Paola Giliberti, Alessandra Santisi, Claudia Columbo, Ludovica Martini, Maria Paola Ronchetti, Paolo Maria Schingo, Guglielmo Salvatori, Fabio Fusaro, Pietro Bagolan, Andrea Dotta, Irma Capolupo, Andrea Conforti

**Affiliations:** 1 Neonatal Intensive Care Unit, “Bambino Gesù” Children’s Hospital IRCCS, 00165 Rome, Italy; francesca.landolfo@opbg.net (F.L.); flaminia.pugnaloni@opbg.net (F.P.); paola.giliberti@opbg.net (P.G.); alessandra.santisi@opbg.net (A.S.); claudia.columbo@opbg.net (C.C.); ludovica.martini@opbg.net (L.M.); mariapaola.ronchetti@opbg.net (M.P.R.); guglielmo.salvatori@opbg.net (G.S.); andrea.dotta@opbg.net (A.D.); irma.capolupo@opbg.net (I.C.); 2 PhD Course in Microbiology, Immunology, Infectious Diseases, and Transplants (MIMIT), Faculty of Medicine and Surgery, “Tor Vergata” University of Rome, 00133 Rome, Italy; 3 Emergency Imaging Unit, “Bambino Gesù” Children’s Hospital IRCCS, 00165 Rome, Italy; pmsalvatore.schingo@opbg.net; 4 Neonatal Surgery Unit, “Bambino Gesù” Children’s Hospital IRCCS, 00165 Rome, Italy; fabio.fusaro@opbg.net (F.F.); pietro.bagolan@opbg.net (P.B.); andrea.conforti@opbg.net (A.C.); 5 Department of Systems Medicine, University of Rome “Tor Vergata”, 00165 Rome, Italy

**Keywords:** meconium obstruction, Fluimucil, intestinal obstruction, acute abdomen

## Abstract

(1) Background: The use of N-acetylcysteine (NAC) to relieve meconium obstruction of prematurity in the first days of life has been reported, with NAC reducing the viscosity of luminal contents by cleaving the disulfide bonds of mucoproteins. However, its use in this population should be further explored since it has been associated with hypernatremia and transient increase in transaminases and bilirubin. (2) Methods: In this retrospective study, we included neonates admitted because of enteral feeding intolerance and intestinal obstruction from 2019 to 2021 who received NAC as a rescue therapy before explorative laparotomy. (3) Results: We summarized the clinical presentation of six preterm neonates with enteral feeding intolerance and intestinal obstruction who received NAC as a rescue therapy. Four infants (66.7%) gradually improved without the need for explorative laparotomy, whereas two infants (33.3%) underwent the creation of an ileostomy. No cases of hypernatremia or hepatic derangement associated with NAC therapy were observed. (4) Conclusions: We described the use of NAC treatment by nasogastric tube and/or rectal enemas in preterm infants with enteral feeding intolerance and intestinal obstruction after a multidisciplinary assessment, but the limited sample size did not allow us to obtain definitive conclusions and further research is needed in this field, given the limited evidence about NAC treatment in preterm infants.

## 1. Introduction

Intestinal obstruction in the neonate could be due to different conditions, including non-surgical conditions (such as the physiological intestinal immaturity of the preterm infant) [1] and surgical conditions (such as intestinal atresia and stenosis, an annular pancreas, malrotation, a duplication cyst, Hirschsprung’s disease, or other causes or situations in which surgery is not always necessary) [2].

N-acetylcysteine (NAC) is used to treat meconium ileus in neonates [3] and distal intestinal obstruction syndrome (DIOS) in older children, especially those with cystic fibrosis [4]. The use of NAC to relieve meconium obstruction of prematurity in the first days of life has been reported, with NAC reducing the viscosity of luminal contents by cleaving disulfide bonds of mucoproteins [5]. Its use in this population should be further explored since it has been associated with hypernatremia and a transient increase in transaminases and bilirubin [3,6].

The aim of our case series was to describe our experience in the use of NAC in preterm neonates with enteral feeding intolerance and intestinal obstruction.

## 2. Materials and Methods

### 2.1. Study Design

This retrospective study was performed in the III-level Neonatal Intensive Care (NICU) of “Bambino Gesù” Children’s Hospital in Rome (Italy), including neonates admitted because of enteral feeding intolerance and intestinal obstruction from the 1st of January 2019 to the 31st of December 2021 who received NAC as a rescue therapy before explorative laparotomy.

All infants had enteral feeding intolerance, defined as the inability to digest enteral feedings associated with increased gastric residuals (of more than 50% of the meal or of a smaller quantity but with the presence of bile) and abdominal distension, although they received a continuous administration of meals via nasogastric tube. Surgical anomalies such as intestinal atresias and necrotizing enterocolitis were radiologically excluded.

Abdominal distension was documented by serial abdomen X-ray examinations, with images compatible with intestinal obstruction.

We included both infants who had benefited from NAC and infants who required ileostomy despite NAC treatment.

We extracted data from medical records, collecting details about enteral feeding intolerance, results of abdomen X-rays, modality of NAC administration, changes in enteral feeding tolerance, and/or the need for explorative laparotomy.

### 2.2. Use of NAC as Rescue Therapy

We used, as a rescue therapy before a possible surgical exploration, a NAC solution (Fluimucil^®^, Zambon, Italy), injecting 1 mL/kg/day of acetylcysteine 4% (diluted in sodium chloride 0.9%) by nasogastric tube and/or by rectal enema.

### 2.3. Ethical Approval

This study reports only a retrospective analysis of data available through the Institutional Database. Since it was conducted on the patients’ medical records, it did not require the approval of our Ethics Committee, which was, however, notified, according to the respective legislation concerning retrospective scientific studies. Personal data were restricted to essential information and were treated in order to guarantee the respect of the privacy of the involved patients, as specifically stated by Italian Law D. Lgs. n.196 of 2003 about personal data protection. Parents of included patients were informed of the therapeutic procedures necessary for their child upon admission to the Neonatal Intensive Care Unit, including those related to the laparotomy, and they gave their consent to the procedures and to the use of clinical data of their children for research purposes.

### 2.4. Review of Literature

In order to review the literature about the use of N-acetylcysteine in preterm neonates with enteral feeding intolerance and intestinal obstruction, an extensive literature search in the MEDLINE database (via PubMed) was performed up to 20 June 2024. The following keywords “N-acetylcysteine” OR “Fluimucil” AND “preterm neonate” OR “preterm newborn” were searched as entry terms as well. All 1095 retrieved articles were screened by reading the abstract; if the abstract reported the administration of NAC, we assessed the full text for inclusion. References in the relevant papers were also reviewed, and further articles were added if necessary. Papers written in languages other than English were excluded. Available information was systematically collected.

## 3. Results

During the study period, six preterm neonates with enteral feeding intolerance and intestinal obstruction who received NAC as a rescue therapy were admitted to our NICU. The characteristics of the neonates are listed in Table 1.

Five infants (83.3%) received NAC both orally and via enema, while one infant (16.7%, P5) received only NAC enemas. Before NAC administration, five patients (83.3%, P1–P5) received human milk, whereas only patient P6 received hydrolyzed formula. Four infants (66.7%, P2–P5) gradually improved without the need for explorative laparotomy, whereas two infants (33.3%, P1 and P6) underwent the creation of an ileostomy. No cases of hypernatremia or hepatic derangement associated with NAC therapy were observed.

### 3.1. Patient 1 (P1)

A female infant was born to a 42-year-old primipara, with pregnancy achieved via medically assisted reproduction and complicated by intrauterine growth restriction (IUGR). An emergency cesarean section was performed at 29 weeks of gestational age (GA) because of both gestational hypertension and non-reassuring fetal heart tracing. The birthweight was 598 g (−2.21 SDS, small for the gestational age). The Apgar scores were 7 and 8 at 1 and 5 min, respectively.

The baby received a first dose of surfactant and was rapidly extubated on the 1st day of life (dol). After meconium passage (achieved within 1st dol), she underwent minimal enteral nutrition (MEN) in the 2nd dol. On the 7th dol, she received a two-day course of paracetamol (15 mg/kg four times/day) to facilitate the closure of the patent ductus arteriosus (PDA) without success. On the 8th dol, the baby developed symptomatic anemia and coagulopathy requiring fresh frozen plasma and red blood cell transfusions. Prominent abdominal distention was observed on the 10th dol along with severe feeding intolerance and intestinal distension. Because of her worsening respiratory status, she needed reintubation and mechanical ventilation. The baby was therefore referred to our NICU for surgical evaluation.

**Figure 1:**
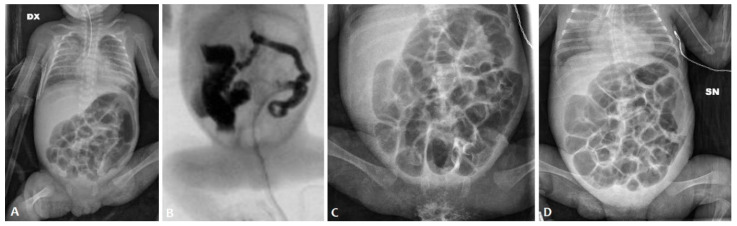
Abdominal X-ray of patient P1 on the 10th day of life (**A**); contrast enema performed on the 26th day of life (**B**); abdominal X-ray before the NAC treatment (**C**) and after the NAC treatment (**D**).

At admission (weight 650 g), an abdominal X-ray (AXR) confirmed the presence of a diffuse dilated bowel with no evidence of intra-abdominal free air (Figure 1A), and an abdominal ultrasound revealed no signs of pneumatosis. Relevant abdominal distention persisted at AXR for the next 15 days despite enteral fasting; during this period, there was no spontaneous stool production. A contrast enema was performed on day 26 using the nonionic dimer contrast agent iodixanol 320 (Visipaque), and it showed a diffuse reduction in colon caliber with multiple fecal residues (Figure 1B).

Thus, the patient received a 15-day course of NAC by nasogastric tube (twice a day) and rectal enemas (once daily). This treatment protocol resulted in an increase in mean daily stool frequency and consistency. AXR findings before and after the NAC treatment course are shown in Figure 1C,D.

Besides the effect of NAC on stool frequency, significant abdominal distension persisted, and an explorative laparotomy was performed on the 42nd dol at a weight of 1112 g. The small bowel was carefully examined and appeared intact and well-perfused. There was no evidence of necrotizing enterocolitis (NEC) or intestinal pathology on gross examination. Loop ileostomy was performed at 10 cm distal to the ileocecal valve. Enteral feeding was restarted on day 15 after ileostomy without abdominal distension recurrence. The ileostomy was closed on the 123rd dol (weight: 2000 g), and the child was discharged home with full enteral feeding on the 134th dol, weighing 2160 g.

### 3.2. Patient 2 (P2)

A female infant was born via emergency cesarean section because of placental abruption at 24 weeks of GA. The pregnancy was physiological, and no maternal pathologies or other prenatal problems were reported. The birthweight was 650 g (+0.33 SDS, appropriate for the gestational age). The Apgar scores were 5 and 6 at 1 and 5 min, respectively. At a few hours old, she was intubated, and surfactant was administered endotracheally because of respiratory distress. Echocardiography revealed a hemodynamically significant patent ductus arteriosus (hs-PDA), for which different attempts at pharmacological closure (ibuprofen, paracetamol, and subsequently indomethacin) were performed without success; the need for mechanical ventilation persisted. Minimal enteral feeding was initiated at 72 h of life, but the baby was fasted because of abdominal distension. The infant did not have a delayed emission of meconium.

**Figure 2:**
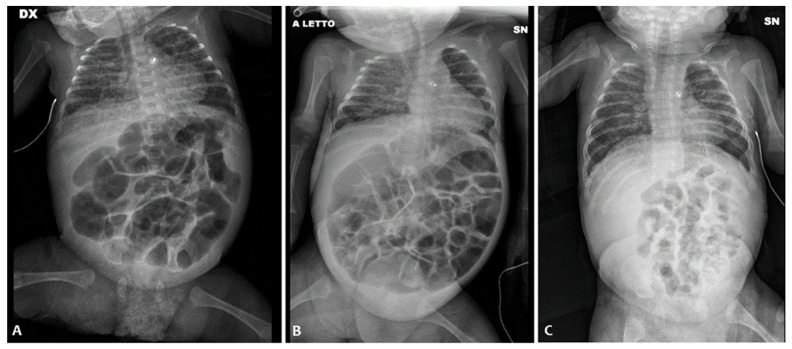
Abdominal X-ray of patient P2 on the 36th day of life (**A**); persistent abdominal distension on the 86th day of life (**B**); and abdominal X-ray after the NAC treatment (**C**).

She was then transferred to our NICU to undergo percutaneous closure of the PDA performed at around 34 days of life, in the absence of postoperative complications. Despite the closure of the PDA, the subsequent attempt at enteral feeding was followed by abdominal distension initially attributed to reperfusion syndrome. Abdominal ultrasonography revealed no signs of pneumatosis and no intestinal and biliary tract abnormalities. Serial abdomen X-ray exams showed diffuse intestinal dilatation and no evidence of intra-abdominal free air (Figure 2A). Relevant abdominal distention persisted, and during this period, there was no spontaneous stool production. A contrast enema was performed using the nonionic dimer contrast agent iodixanol 320 (Visipaque) and showed dolichosigma exclusively. Barium enema and small bowel enema studies were performed, with normal results. Newborn metabolic screening and genetic tests for cystic fibrosis were negative. We found no hormone anomalies.

At around 56 days of life, in the suspicion of an intestinal motility disorder, therapy was started by nasogastric tube with amoxicillin–clavulanic acid at a dosage of 10 mg/kg twice a day, and at the same time, NAC rectal enemas were started twice a day. The execution of the enemas determined the emission of stools (normal color and consistency) but without improvement in abdominal distension.

On the 86th day of life (Figure 2B), postponed due to an episode of sepsis caused by Serratia marcescens, minimal enteral feeding (using breast milk) was started in association with the administration of NAC via a nasogastric tube. With the introduction of NAC treatment, there was a regularization of the frequency and consistency of the stools andan absence of clinically relevant abdominal distension (Figure 2C), which made it possible to reach full enteral feeding and weight gain. The infant was discharged home with full oral feeding on day 110.

### 3.3. Patient 3 (P3)

A male infant was born to a mother with systemic erythematous lupus and preeclampsia, in treatment with prednisone and methyldopa. An emergency cesarean section was performed at 30 + 3 weeks of GA because of maternal hypertension and IUGR. The birthweight was 1350 g (−0.07 SDS, appropriate for the gestational age). The Apgar score was 3 at the 1st minute, and he required endotracheal surfactant administration due to a severe cardiorespiratory failure. At the referring center, a rectal enema with NAC was administered at four days of life because of failure to pass meconium, with consequently delayed passage of scarce traces of stool.

Afterward, on the 4th dol, the baby was intubated again and then transferred to our NICU because of worsening abdominal distension; in the suspicion of intestinal obstruction or NEC, antibiotic treatment was started.

**Figure 3:**
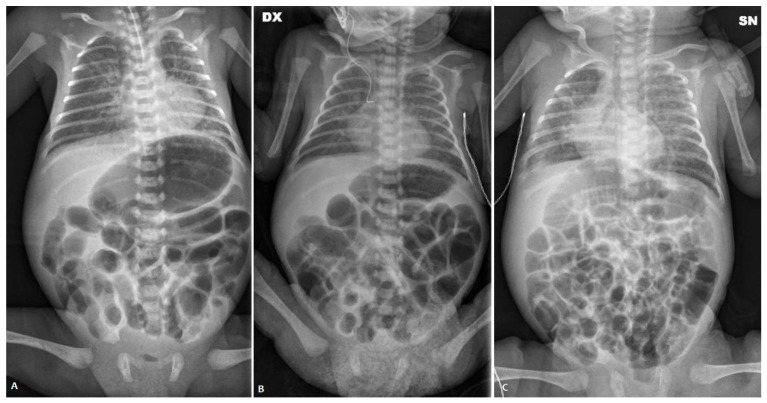
Abdominal X-ray of patient P3 on the 3rd day of life (**A**); abdominal X-ray before the NAC treatment (**B**) and after the NAC treatment (**C**).

At admission, the belly was distended (Figure 3) and painful on palpation, with bile-stained vomiting. AXR showed no signs of NEC. On the 5th day of life, after a rectal enema with saline solution, a sticky meconium passage was observed. Therefore, NAC rectal enemas were performed daily. On the 8th day of life, NAC was also given by nasogastric tube (three times/day). Enteral feeding was started on the 13th day of life and then transiently stopped because of two septic episodes. Cystic fibrosis and thyroid anomalies were ruled out. Full enteral feeding was reached on the 80th day of life and NAC rectal enemas were stopped.

### 3.4. Patient 4 (P4)

**Figure 4:**
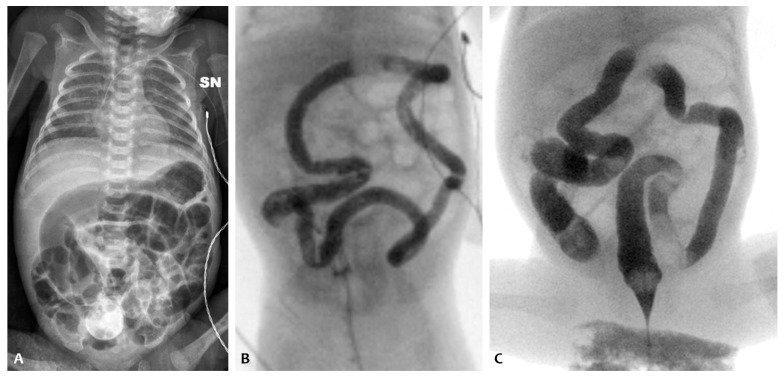
Abdominal X-ray of patient P4 on the 8th day of life (**A**); contrast enema before the NAC treatment (**B**) and after the NAC treatment (**C**).

A female neonate was born after a preterm premature rupture of membranes longer than 24 h. The mother received both systemic antibiotics and steroid prophylaxis. An emergency cesarean section was performed at 26 weeks + 3 days of GA because of a non-reassuring fetal heart tracing. The birthweight was 870 g (+0.40 SDS, appropriate for the gestational age). The Apgar score was 5 at the 1st minute and then she needed intubation, receiving two surfactant doses afterward. The patency of the ductus arteriosus was successfully treated using two doses of paracetamol. She did not pass meconium either spontaneously or after enemas during the first 72 h of life. A minimal enteral feeding with donor human milk was started within the 3rd day of life, but she presented biliary gastric residuals, abdominal distension, and vomiting. An abdomen contrast X-ray performed on the 8th day of life revealed the presence of intestinal obstruction (Figure 4A). Therefore, she was referred to our NICU for a surgical evaluation.

A lower contrast enema was performed on the 10th day of life using the non-ionic dimer contrast agent iodixanol 320 (Visipaque^®^), revealing a diffuse reduction in colon caliber with multiple fecal residues (Figure 4B). The patient received a 15-day course of NAC by nasogastric tube (twice a day) and rectal enemas (once daily). This treatment protocol resulted in an increase in mean daily stool frequency and consistency. Minimal enteral feeding was started again on the 24th day of life with good tolerance and progressively increased, reaching full enteral feeding on the 57th day of life.

### 3.5. Patient 5 (P5)

A male infant was delivered via emergency cesarean section at 32 + 6 weeks of gestational age due to maternal gestational hypertension. The birthweight was 1490 g (−0.81 SDS, appropriate for the gestational age). The Apgar score was 7 at the 1st minute and 9 at the 5th minute, respectively. He required nCPAP for 24 h, and then he spontaneously breathed. He passed meconium on the first day of life, and minimal enteral feeding was given only for three meals because of abdominal distension.

**Figure 5:**
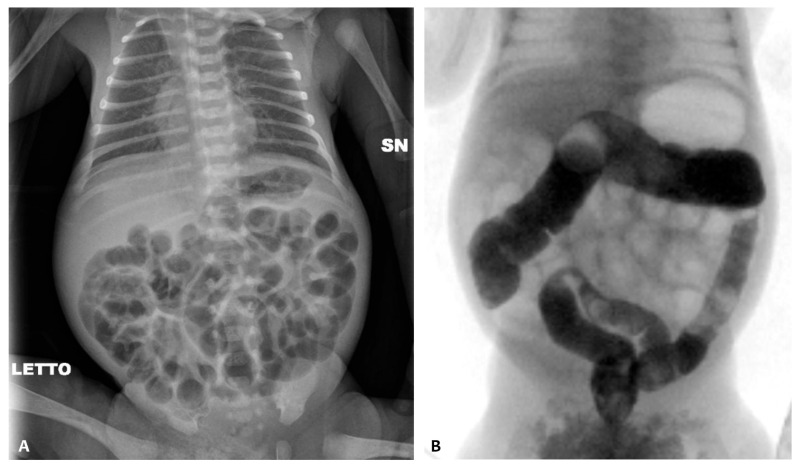
Abdominal X-ray of patient P5 on the 6th day of life (**A**); barium enema before the NAC treatment (**B**).

He was referred to our NICU for a surgical evaluation on the 6th day of life (X-ray shown in Figure 5A). The abdomen was treatable and non-tender to palpation, with visibly dilated intestinal loops and a well-reducible left inguinal swelling. After rectal probing, a leakage of normocolic sticky stools was observed.

A barium enema was performed the day after, with radiological evidence of meconium residues without interruption of the contrast medium (Figure 5B). The baby underwent NAC rectal enemas once daily with clinical improvement of abdominal distension. From the 8th day of life, he gradually recovered and well tolerated enteral feeding, reaching full enteral feeding on the 22nd day of life.

However, due to the persistent need for rectal enemas to evacuate, rectal biopsies were performed by suction. Classical forms of congenital aganglionic megacolon were ruled out. The hormone profile was also normal.

### 3.6. Patient 6 (P6)

A male infant was born via emergency cesarean section because of placenta previa and chorioamnionitis at 23 weeks of GA, after a twin pregnancy obtained by medically assisted reproduction. Chorionic villus sampling revealed no chromosomic anomalies. The birthweight was 710 g (+1.57 SDS, large for the gestational age). The Apgar scores were 1 and 6 at 1 and 5 min, respectively. At birth, he was intubated because of severe respiratory failure and then required different doses of ibuprofen and indomethacin to close the hs-PDA. Enteral feeding was started at 7 days of life, but it was never well tolerated. Indeed, during his NICU stay, he presented with abdominal distension and partial intestinal occlusion, treated with NAC by nasogastric tube and with rectal enemas of sodium saline, but only with partial improvement of enteral feeding tolerance and abdominal distension.

He was referred to our NICU at the postmenstrual age of 34 weeks because of retinopathy of prematurity. He still required oxygen therapy and presented with cholestatic jaundice. Genetic causes of liver disorders were ruled out.

**Figure 6:**
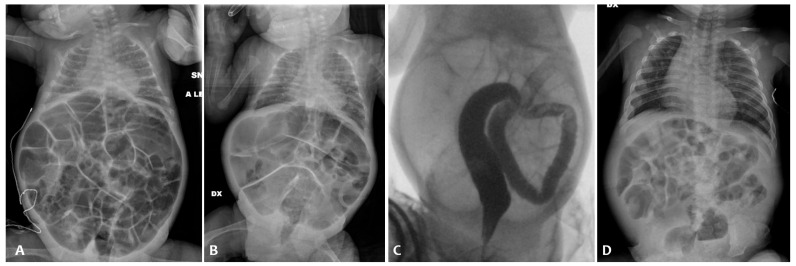
Abdominal X-ray of patient P6 at 34 weeks of postmenstrual age at our NICU admission (**A**) and after NAC rectal enemas (**B**); barium enema documenting dolichosigma and intestinal malrotation (**C**); and abdominal X-ray at discharge (**D**).

A second episode of intestinal sub-occlusion occurred, with diffuse dilated intestinal loops at the abdomen X-ray (Figure 6). NAC rectal enemas were given. Because of the lack of clinical improvement, a barium enema was performed, documenting a dolichosigma, with a regular caliber of the whole colon and without caliber changes, and a medialized cecum and colon in relation to the marked distension of the small bowel, with an overall picture of intestinal malrotation. Cystic fibrosis was ruled out via genetic sequencing and a sweat test.

An ileostomy was performed at 10 cm from the ileocecal valve, with the resolution of obstructive symptoms and enteral tolerance. The stoma was closed after 2 months. He was discharged home at the corrected age of around 3 months.

### 3.7. Review of the Literature

As shown in Table 2, we summarized the available literature (including our cohort) on the use of NAC in preterm neonates with enteral feeding intolerance and intestinal obstruction. Beyond some local guidelines, only six articles besides ours cover this treatment for this condition [3,5,7,8,9,10].

## 4. Discussion

Preterm neonates may have both medical and surgical conditions that cause intestinal obstructive symptoms. When there is a relative indication for surgery, whether a case can be conservatively managed or whether it needs surgical exploration continues to remain a debatable issue [11,12,13,14,15,16,17].

An accurate differential diagnosis of the possible causes of intestinal obstruction should be performed, keeping in mind that preterm patients are already at high risk of hemodynamic instability and neurocognitive developmental issues when undergoing surgery [18,19]. Considering these alerts coming from the literature, surgery should be avoided, if possible. In addition to its anti-inflammatory and antioxidant effects, which favor the maintenance of a cellular redox imbalance [20], NAC is used as first-line treatment in newborns with functional obstruction related to inspissated meconium, such as meconium ileus in cystic fibrosis, milk curd obstruction, etc. [3,4,5,6,9], through an osmotic gradient that promotes the retrieval of water.

In this retrospective study, we describe a possible use of N-acetylcysteine in preterm neonates with enteral feeding intolerance and intestinal obstruction. Beyond its use in cystic fibrosis, NAC treatment for neonatal meconium obstruction has long been reported, even in very low birthweight infants [7,21], but it is still not routinely used in all NICUs. Neonatologists should be more aware of the possible use of this drug in non-surgical cases of functional intestinal obstruction. Watchful waiting is usually preferred in asymptomatic infants, whereas an intervention is required in symptomatic infants, i.e., those with feeding intolerance or abdominal distension.

Pure NAC is hyperosmolar (2600 mOsmol/L) and, therefore, a dilution is suggested (using water for injection or saline solution) [22]; however, the administered amount is so low that even a more concentrated solution would have a low osmolarity.

Garza-Cox et al. previously described the use of NAC in symptomatic, very low-birthweight infants with meconium obstruction, but only in the postoperative period [7].

Ozdemir et al. described the antioxidant effects of NAC in a neonatal rat model of necrotizing enterocolitis; intestinal tissue tumor necrosis factor α levels were significantly reduced in mice treated with NAC rather than in those treated with saline solution only [23].

Furthermore, the literature suggests that Toll-like receptor 4 (TLR-4) is expressed at higher levels in the premature intestine and that it is more abundant in the intestine of infants with NEC through the modulation of the epithelial cell barrier and regulation of the innate and adaptive immune responses [24,25]. Interestingly, Beloosesky et al. reported the role of intestine TLR-4 modulation following N-acetyl-cysteine treatment in a NEC rodent model. When NAC was administered in rats, they observed a decrease in ileum TLR-4 levels and an increase in ileum glutathione levels (with their anti-inflammatory properties) [26]. Given that oxidative stress and inflammatory mediators contribute to the pathogenesis of NEC, NAC could have a protective effect on intestinal injury through its anti-inflammatory and antioxidant properties.

All of these six cases were similar in persistent enteral feeding intolerance and the radiological characteristics of abdominal obstruction; all six patients were born with a very low birthweight and were referred to our center at a postmenstrual age ranging from 30 to 34 weeks (except for patient P4 at 27 weeks). In most cases, human milk was used for enteral feeding before NAC. Four patients improved with NAC administration, whereas only two patients required the creation of an ileostomy. Human milk was given after NAC administration in all cases (in particular, donor human milk in patient P6 due to the lack of his own’s mother milk).

NAC was given for a median length of 26 days (IQR 18–48). The small sample size prevents us from drawing definitive conclusions regarding the duration of treatment; patients who underwent ileostomy received NAC for approximately 20 days, while the others received a more prolonged treatment (median: 40 days, IQR: 21–55) when a clinical improvement was observed. In these fragile patients, the achievement of full enteral feeding may be influenced by many factors, such as the onset of late-onset sepsis, the duration of antibiotic therapy, and the duration of fasting periods.

We detected no NAC-related side effects and, above all, no hypernatremia or liver injury.

Obviously, the retrospective study design has its limitations, with the limited possibility of generalizing the validity of these results and the impossibility of establishing a cause–effect relationship. We included only six infants referred to our surgical reference center in a 3-year period, and therefore, an inclusion bias could be present; preterm infants with milder symptoms from referring centers may have been excluded. We hope to shed light on this issue in future multicenter randomized controlled trials. However, it is difficult to perform a randomized controlled trial in highly selected cases such as these; all these cases were outborn and came from non-surgical NICUs after different kinds of management. Particular caution is required, considering that a persistent obstruction can lead to intestinal perforation. Therefore, the still-limited evidence about NAC treatment in preterm infants with intestinal obstruction prevents us from advising its use without a surgical evaluation, and without knowledge of the timing within which the effects of the drug can manifest themselves.

## 5. Conclusions

We described the use of NAC treatment by nasogastric tube and/or rectal enemas in preterm infants with enteral feeding intolerance and intestinal obstruction after a multidisciplinary assessment involving neonatologists, radiologists, and neonatal surgeons in the decision-making. In these children, we observed no complications that outweigh the benefits of use. However, NAC treatment is not a magic wand; in some neonates with particular intestinal anatomy, only the creation of an ileostomy resolved the clinical picture of enteral feeding intolerance and intestinal pseudo-obstruction.

Therefore, further research is needed in this field to verify in which preterm neonates surgical exploration could be avoided by a NAC treatment.

## Figures and Tables

**Table 1:** Descriptive information concerning the six patients with intestinal obstruction.

Demographics	Patients (*n* = 6)
Gestational age at birth, weeks—median (IQR)	27.7 (24.8–30.0)
Birthweight, grams—median (IQR)	790 (665–1230)
Postmenstrual age at referral, weeks—median (IQR)	32.0 (30.5–33.3)
Weight at referral, grams—median (IQR)	1225 (926–1498)
Medically assisted reproduction, *n* (%)	1 (16.7%)
Males, *n* (%)	3 (50.0%)
Postmenstrual age at NAC, weeks—median (IQR)	32.2 (30.5–33.3)
Enteral fasting, days—median (IQR)	18.5 (11.5–40.5)
Parenteral nutrition, days—median (IQR)	77.5 (57.3–86.5)
Central lines duration, days—median (IQR)	85.0 (76.0–89.5)
Sepsis episodes, median (IQR)	2.5 (1.5–3.0)
Antibiotic duration, days—median (IQR)	35.5 (32.8–38.3)

**Table 2:** Available reports about the use of N-acetylcysteine in preterm neonates with enteral feeding intolerance and intestinal obstruction to date.

	First Author, Year	Type of Study	Number of Included Patients Managed with NAC	Methods of NAC Administration	Summary
1	Shaw, 1969 [5]	Experimental animal study about intestinal mucosa exposed to NAC	48 puppies from 3 days to 3 months of age	NAC enema at different concentrations (4%, 10%, and 20%)	The 20% and 10% NAC solutions are not safe, whereas the 4% NAC solution is effective but had no adverse effects on the mucosal histology
2	Langer et al., 1990 [3]	Case report	1 extremely low-birthweight	Instillation of 5% NAC into the distal ileum through the ileostomy	They reported hypernatremia associated with the used NAC formulation; the authors reported that using another formulation, they observed no changes in the serum sodium concentrations
3	Garza-Cox et al., 2004 [7]	Retrospective clinical study	1 extremely low-birthweight preterm infant	20% NAC enema, after an unsuccessful saline enema	Pneumoperitoneum was evident at X-ray immediately after instillation of NAC enema; he underwent laparotomy: two intestinal perforations and inspissated meconium were found, requiring ileostomy
4	Solaz-García et al., 2019 [8]	Retrospective observational study	6 very low-birthweight preterm infants	NAC through an orogastric tube (not reported the concentration) and Gastrografin^®^ enemas, if physiological serum enema was not effective in solving delay in meconium expulsion	Conservative treatment seems to be an effective therapeutic measure for the prevention of meconium obstruction in very low-birthweight preterm infants since it achieved the expulsion of meconium without having to apply surgical treatment
5	Simsek et al., 2019 [9]	Retrospective observational study	121 very low-birthweight preterm infants	34 infants received oral 10% NAC (group 1), 52 received rectal 10% NAC (group 2), and 35 served as a control group (group 3)	They compared, for the first time, rectal and oral NAC in preterm infants with meconium ileus and did not observe any differences between the two groups in reaching full enteral feeding
6	Gross et al., 2022 [10]	Survey of current practice for promoting meconium passage in Germany	Very preterm infants (not reported specific cases)	Orally and/or enema	NAC was the agent used in 25% of centers, singularly or in association with glucose or normal saline. Other centers used other methods (normal saline, contrast agent, glycerin, glucose 5%, lipid solution, breast milk, maltodextrins).
7	De Rose et al., 2024	Case series	6 very low-birthweight preterm infants who received NAC	Orally and/or enema 4% NAC	In some cases, NAC can solve intestinal obstruction and avoid surgical intervention; in other cases, only the creation of an ileostomy solves the clinical picture.

## Data Availability

All data considered for this case report have been included in this article. Articles considered for the review of literature are already available on PubMed.

## References

[1] Healy D.B., Ryan C.A., Ross R.P., Stanton C., Dempsey E.M. (2022). Clinical Implications of Preterm Infant Gut Microbiome Development. Nat. Microbiol..

[2] Hajivassiliou C.A. (2003). Intestinal Obstruction in Neonatal/Pediatric Surgery. Semin. Pediatr. Surg..

[3] Langer J.C., Paes B.M., Gray S. (1990). Hypernatremia Associated with N-Acetylcysteine Therapy for Meconium Ileus in a Premature Infant. Can. Med. Assoc. J..

[4] Schauble A.L., Bisaccia E.K., Lee G., Nasr S.Z. (2019). N-Acetylcysteine for Management of Distal Intestinal Obstruction Syndrome. J. Pediatr. Pharmacol. Ther..

[5] Shaw A. (1969). Safety of N-Acetylcysteine in Treatment of Meconium Obstruction of the Newborn. J. Pediatr. Surg..

[6] Cooke A., Deshpande A.V., Wong C.K.F., Cohen R. (2008). Hepatic Derangement Following N-Acetylcysteine Enemas in an Infant with Cystic Fibrosis. J. Paediatr. Child Health.

[7] Garza-Cox S., Keeney S.E., Angel C.A., Thompson L.L., Swischuk L.E. (2004). Meconium Obstruction in the Very Low Birth Weight Premature Infant. Pediatrics.

[8] Solaz-García A.J., Segovia-Navarro L., Rodríguez de Dios-Benlloch J.L., Benavent-Taengua L., Castilla-Rodríguez D.Y., Company-Morenza M.A. (2019). Prevention of Meconium Obstruction in Very Low Birth Weight Preterm Infants. Enfermería Intensiv. (Engl. Ed.).

[9] Simsek G.K., Arayici S., Buyuktiryaki M., Okur N., Kanmaz Kutman G., Oguz S.S. (2019). Oral N-Acetyl Cysteine for Meconium Ileus of Preterm Infants. Gynecol. Obstet. Reprod. Med..

[10] Gross M., Hummler H., Haase B., Quante M., Wiechers C., Poets C.F. (2022). Interventions for Promoting Meconium Passage in Very Preterm Infants—A Survey of Current Practice at Tertiary Neonatal Centers in Germany. Children.

[11] de la Hunt M.N. (2006). The Acute Abdomen in the Newborn. Semin. Fetal Neonatal Med..

[12] Longardt A.C., Loui A., Bührer C., Berns M. (2019). Milk Curd Obstruction in Human Milk-Fed Preterm Infants. Neonatology.

[13] De Rose D.U., Romano V., Priolo F., Zecca C., Maggio L., Vento G., Gallini F. (2021). An Unusual Pneumoperitoneum in an Extremely Low Birth Weight Preterm Newborn. Acta Biomed..

[14] Basany L., Aepala R., Bellary M.M.R., Chitneni M. (2015). Intestinal Obstruction Due to Ileal Duplication Cyst and Malrotation in a Preterm Neonate. J. Neonatal Surg..

[15] Tolaymat Y., Irons R., Taylor J.A., Rajderkar D., Cacho N. (2023). Does Preterm Status Hinder the Timely Diagnosis of Intestinal Atresia?. Neoreviews.

[16] Verma A., Rattan K.N., Yadav R. (2016). Neonatal Intestinal Obstruction: A 15 Year Experience in a Tertiary Care Hospital. J. Clin. Diagn. Res..

[17] Desoky S., Kylat R., Udayasankar U., Gilbertson-Dahdal D. (2018). Managing Neonatal Bowel Obstruction: Clinical Perspectives. Res. Rep. Neonatol..

[18] McCann M.E., Schouten A.N.J. (2014). Beyond Survival; Influences of Blood Pressure, Cerebral Perfusion and Anesthesia on Neurodevelopment. Paediatr. Anaesth..

[19] Lo E., Kalish B.T. (2023). Neurodevelopmental Outcomes after Neonatal Surgery. Pediatr. Surg. Int..

[20] Tenório M.C.D.S., Graciliano N.G., Moura F.A., de Oliveira A.C.M., Goulart M.O.F. (2021). N-Acetilcysteine (NAC): Impacts on Human Health. Antioxidants.

[21] Emil S., Nguyen T., Sills J., Padilla G. (2004). Meconium Obstruction in Extremely Low-Birth-Weight Neonates: Guidelines for Diagnosis and Management. J. Pediatr. Surg..

[22] Villeneuve E., Gosselin S., Brent J., Burkhart K., Dargan P., Hatten B., Megarbane B., Palmer R. (2017). N-Acetylcysteine. Critical Care Toxicology: Diagnosis and Management of the Critically Poisoned Patient.

[23] Ozdemir R., Yurttutan S., Sari F.N., Uysal B., Unverdi H.G., Canpolat F.E., Erdeve O., Dilmen U. (2012). Antioxidant Effects of N-Acetylcysteine in a Neonatal Rat Model of Necrotizing Enterocolitis. J. Pediatr. Surg..

[24] Nanthakumar N., Meng D., Goldstein A.M., Zhu W., Lu L., Uauy R., Llanos A., Claud E.C., Walker W.A. (2011). The Mechanism of Excessive Intestinal Inflammation in Necrotizing Enterocolitis: An Immature Innate Immune Response. PLoS ONE.

[25] Gomart A., Vallée A., Lecarpentier Y. (2021). Necrotizing Enterocolitis: LPS/TLR4-Induced Crosstalk Between Canonical TGF-β/Wnt/β-Catenin Pathways and PPARγ. Front. Pediatr..

[26] Beloosesky R., Gutzeit O., Ginsberg Y., Khatib N., Ross M.G., Weiner Z., Zmora O. (2023). Intestine and Brain TLR-4 Modulation Following N-Acetyl-Cysteine Treatment in NEC Rodent Model. Sci. Rep..

